# HIV pre-exposure prophylaxis (PrEP)- knowledge and attitudes among a New York City emergency department patient population

**DOI:** 10.1186/1742-4690-9-S1-P94

**Published:** 2012-05-25

**Authors:** Yvette Calderon, Jason Leider, Ethan Cowan, Christopher Brusalis, Joanne Mantell, Theo Sandfort

**Affiliations:** 1 Albert Einstein College of Medicine, Bronx, New York, USA; 2 Jacobi Medical Center, Bronx, New York, USA; 3 Columbia University, New York, New York, USA

## Background

HIV Pre-exposure Prophylaxis (PrEP), in which HIV-negative individuals receive antiretroviral medications to prevent HIV acquisition, has shown potential as a means to reduce HIV incidence among high-risk persons. The acceptability of PrEP among at-risk persons will strongly impact the effectiveness of PrEP. This study aimed to assess knowledge and attitudes towards PrEP within a demographically-mixed community with high HIV prevalence.

## Materials and methods

A cross-sectional study was conducted with a convenience sample of Emergency Department (ED) patients at two New York City municipal hospitals. Eligible participants completed an anonymous written survey about knowledge and acceptability of PrEP. Means and standard deviations were calculated for continuous variables and proportions for categorical variables. Standard bivariate methods were used to compare acceptability and knowledge by race, ethnicity and gender.

## Results

474 ED patients agreed to participate. The study population was 40.9% male, 40.7% Latino and 38.2% non-Hispanic Black. 7 participants (1.5%) self-identified as MSM. Mean age was 35.3, SD +/- 13.1 years. 66.4% reported inconsistent condom use and 78.4% had previously had an HIV test. 13.3% reported knowledge of either the term “PrEP” or the use of antiretroviral medications to prevent HIV acquisition. More people indicated they were unlikely or extremely unlikely to use PrEP (40.1%) than indicated they would likely take PrEP if available (32.2%). Many (27.7%) were unsure if they would or would not take PrEP. 44.4% thought that individuals would stop using condoms if on PrEP, while 27.0% thought that individuals would continue using them. Some participants (28.4%) incorrectly thought that PrEP needed to be taken only prior to sex. There were no differences in knowledge or acceptability of PrEP by gender. Latinos were more likely (17.6%) than blacks (8.8%) and others (12.4%) to report knowledge of PrEP.

## Conclusions

Potential providers of PrEP must consider limitations in acceptability to this HIV prevention strategy. Future administration of PrEP must incorporate patient education to ensure user understanding of the technology and its potential limitations.

